# Vagus nerve stimulation: a physical therapy with promising potential for central nervous system disorders

**DOI:** 10.3389/fneur.2024.1516242

**Published:** 2024-12-13

**Authors:** Chaoran Wang, Bangqi Wu, Ruolan Lin, Yupei Cheng, Jingjie Huang, Yuyan Chen, Jing Bai

**Affiliations:** ^1^First Teaching Hospital of Tianjin University of Traditional Chinese Medicine/National Clinical Research Center for Chinese Medicine Acupuncture and Moxibustion, Tianjin, China; ^2^Postgraduate School, Tianjin University of Traditional Chinese Medicine, Tianjin, China

**Keywords:** vagus nerve stimulation, central nervous system, clinical application, mechanisms, neurophysiotherapy

## Abstract

The diseases of the central nervous system (CNS) often cause irreversible damage to the human body and have a poor prognosis, posing a significant threat to human health. They have brought enormous burdens to society and healthcare systems. However, due to the complexity of their causes and mechanisms, effective treatment methods are still lacking. Vagus nerve stimulation (VNS), as a physical therapy, has been utilized in the treatment of various diseases. VNS has shown promising outcomes in some CNS diseases and has been approved by the Food and Drug Administration (FDA) in the United States for epilepsy and depression. Moreover, it has demonstrated significant potential in the treatment of stroke, consciousness disorders, and Alzheimer’s disease. Nevertheless, the exact efficacy of VNS, its beneficiaries, and its mechanisms of action remain unclear. This article discusses the current clinical evidence supporting the efficacy of VNS in CNS diseases, providing updates on the progress, potential, and potential mechanisms of action of VNS in producing effects on CNS diseases.

## 1 Introduction

**Figure 1:**
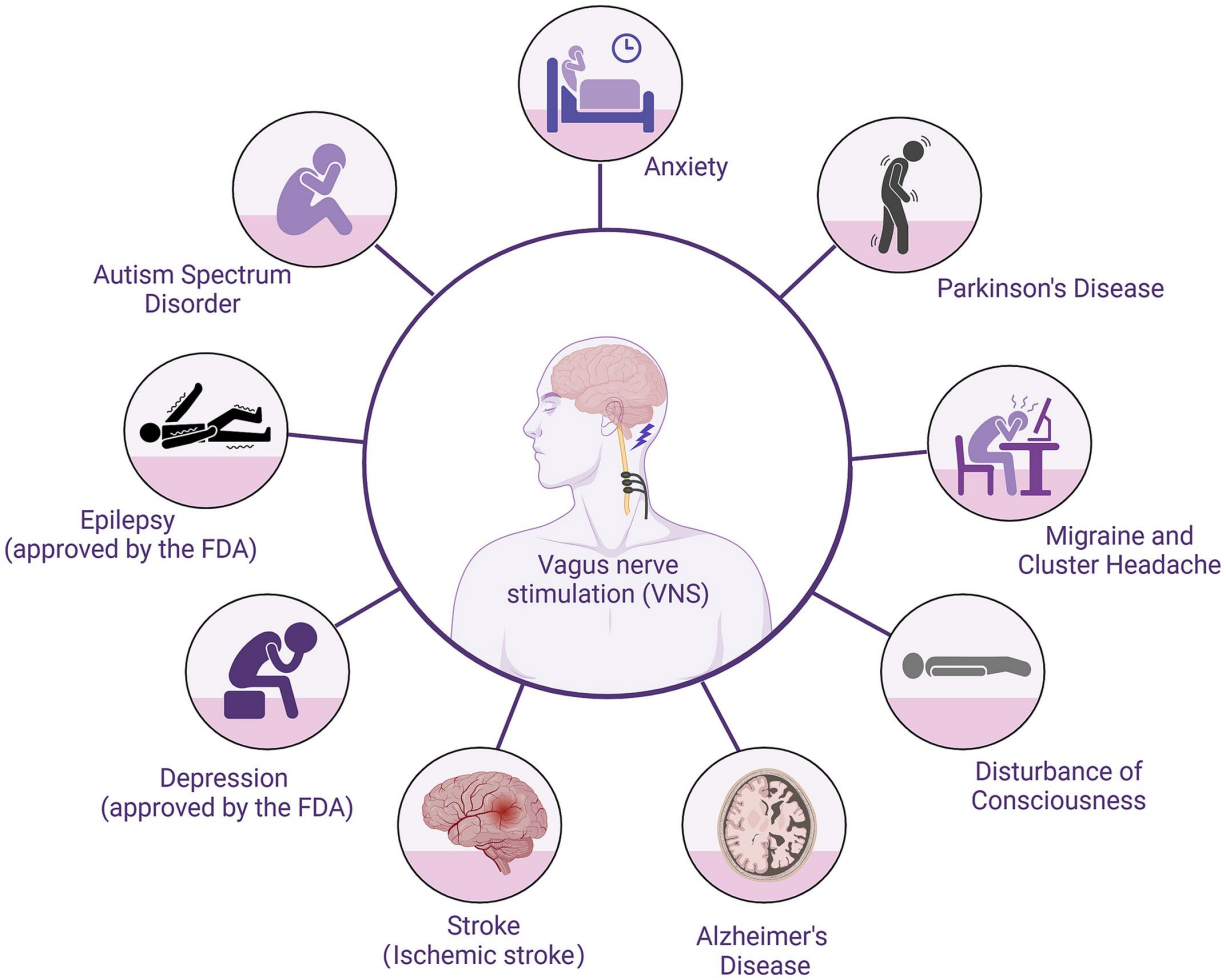
Use of VNS in central nervous system diseases.

The introduction of vagus nerve stimulation into clinical applications has been a long and convoluted process. VNS was initially proposed by American neurologist James Corning in the late 19th century in an attempt to treat epilepsy. Due to his misconceptions about epilepsy, VNS was abandoned (1). It wasn’t until 1988 that the first implantation of VNS was reported (2), leading to an increase in research on vagus nerve stimulation. After years of study, the efficacy and safety of VNS were confirmed, and diseases it could potentially treat gradually emerged. VNS was first approved by the U.S. Food and Drug Administration for treating drug-resistant epilepsy (3). Subsequently, it was later approved for treating refractory depression, obesity, refractory migraines, and cluster headaches (4, 5). With advancements in the technology of implantable vagus nerve stimulation (iVNS) and its associated high surgical costs and side effects, non-invasive vagus nerve stimulation (nVNS) was proposed and implemented. Meanwhile, the clinical applications and effects of iVNS and nVNS also differ. Vagus nerve stimulation is a relatively newer therapeutic approach, and besides its explicitly approved clinical uses, there exist numerous potential applications (Figure 1). This article will provide an overview of the applications, potential, side effects, and mechanisms of action of vagus nerve stimulation in central nervous system diseases.

## 2 Vagus nerve stimulation

### 2.1 Invasive vagus nerve stimulation

Invasive Vagus Nerve Stimulation (iVNS) involves surgically implanting a vagus nerve stimulation device into the human body. The vagus nerve stimulator consists of stimulating electrodes implanted around the vagus nerve through a small cervical incision and a pulse generator implanted in the subclavian region (6, 7). The device delivers electrical stimulation every few minutes. Additionally, patients can use a wristband magnet to trigger the pulse generator, providing an extra dose of stimulation. Over several weeks following implantation, the current is gradually adjusted to the target level based on patient tolerability (7). The electrode types include helical electrodes that wrap around the vagus nerve and intravascular electrodes placed within the internal jugular vein. Helical electrodes are commonly used for broad nerve activation, while intravascular electrodes are designed to reduce off-target effects and enhance stimulation specificity (8). The complications of VNS include those related to surgery and those associated with the device and nerve stimulation. During VNS implantation, the implanted device is typically tested using one pulse train lasting 1 min with specific parameters (1 mA, 550 μs, 20 Hz). This phase may result in bradycardia or even cardiac arrest (6, 9). Surgical complications such as peritracheal hematoma, infection, and vagus nerve injury have also been reported (10). Complications associated with the device and nerve stimulation include delayed arrhythmias (bradycardia, cardiac arrest), laryngopharyngeal dysfunction (hoarseness, dyspnea, and coughing) due to stimulation of the recurrent laryngeal nerve, obstructive sleep apnea, phrenic nerve stimulation caused by proximity, tonsillar pain resembling glossopharyngeal neuralgia, and sternocleidomastoid muscle spasms (6, 11–13). The iVNS therapy system is indicated for stimulating the left vagus nerve located within the carotid sheath, specifically below the point where the superior and inferior cervical cardiac branches diverge from the vagus nerve (14). Typically, the left-sided cervical vagus nerve is chosen as the implantation site for the stimulating electrodes because stimulating the right-sided vagus nerve can lead to severe adverse reactions. The right vagus nerve innervates the sinoatrial node and parts of the myocardium, and nerve stimulation can cause various cardiac side effects, such as cardiac arrest. In comparison to the right-sided vagus nerve, the left-sided vagus nerve innervates the atrioventricular node, resulting in relatively milder adverse reactions (7). Furthermore, studies have shown that stimulation frequencies of 50 Hz or higher during VNS can cause significant and irreversible damage to the vagus nerve. To minimize adverse events associated with direct stimulation of the carotid sheath, stimulation frequencies were randomly selected within the range of 20–30 Hz (14, 15). AspireSR is a pulse generator that detects heart rate through specific detection algorithms, recommended for use in cardiac patients due to the cardiac side effects of VNS (16). Additionally, SenTiva VNS has a cardiac detection feature that not only detects heart activity but also allows for changes in VNS output parameters, making VNS therapy more tailored and safe. The SenTiva device is smaller in size compared to AspireSR. For pediatric patients whose development is not yet complete, having an appropriately sized implantable pulse generator is particularly crucial. Moreover, for those seeking smaller surgical incisions and aesthetic considerations, SenTiva represents a favorable choice. Despite being withdrawn from clinical use by the U.S. Food and Drug Administration in 2020 due to reset errors, SenTiva regained approval in 2022 (16, 17).

### 2.2 Non-invasive vagus nerve stimulation

Invasive Vagus Nerve Stimulation requires surgical implantation, while non-invasive Vagus Nerve Stimulation avoids trauma and is more cost-effective. nVNS devices stimulate the VN at specific body surface sites to achieve therapeutic effects. nVNS comprises transcutaneous auricular Vagus Nerve Stimulation (taVNS) and transcutaneous cervical Vagus Nerve Stimulation (tcVNS), relying on the distribution of vagal afferents in the ear and neck, respectively (18). taVNS transmits signals by stimulating the auricular branch of the vagus nerve (ABVN). Peuker and Filler found anatomical structures in the ear—such as parts of the ear canal and auricle—that send their afferent information via vagal branches. The optimal site for stimulation is the cymba conchae, as ABVN primarily exists in the skin surrounding the cymba conchae (19, 20). tcVNS involves stimulation of the vagus nerve within the carotid sheath, with the stimulation signals activating the VN through the skin and other tissues near the carotid sheath, thereby inducing vagus nerve action potentials. tVNS can be administered using various devices, such as the transcutaneous Vagus Nerve Stimulation device NEMOS and the neck-stimulating gammaCore (21). The use of VNS is restricted to the left vagus nerve, while tVNS might not have this limitation. Research by Chen et al. suggests that unilateral VN stimulation transmits only on one side, while unilateral ABVN stimulation integrates within the nucleus tractus solitarii and then transmits bilaterally via the vagus nerve. Results indicate that right transcutaneous vagus nerve stimulation is as safe as left transcutaneous vagus nerve stimulation (22). Studies by He et al. and Hein E et al. respectively employed bilateral transcutaneous vagus nerve stimulation in clinical trials involving children and adults. No adverse cardiac-related reactions were observed in these experiments (23, 24). Although nVNS is relatively safe, it is not without side effects, which include localized skin irritation caused by electrode placement, headaches, nasopharyngitis, gastrointestinal reactions, palpitations, and vocal cord hoarseness (14). Different devices have varying stimulation methods, frequencies, durations, and other parameters. Currently, there are no standardized optimal stimulation parameters, nor is there literature establishing superiority between taVNS and tcVNS stimulation methods.

## 3 Clinical and potential applications in central nervous system diseases

### 3.1 Epilepsy

Since the 1990s, numerous studies have evidenced the favorable effects of VNS in reducing seizure frequency in epilepsy patients (25, 26). In 1997 and 1999, the American Academy of Neurology (AAN) Therapeutics and Technology Assessment Subcommittee conducted two evaluations of VNS therapy for epilepsy. The committee concluded that VNS is suitable for adults and adolescents aged 12 and above with refractory partial-onset seizures (excluding those suitable for potentially curative surgical resection) (27, 28). Due to the infrequent occurrence of complete seizure remission with VNS and its invasiveness and high cost, VNS is deemed more appropriate for individuals intolerant to or not benefiting from antiseizure medication (ASMs) (28). Subsequently, the feasibility and safety of VNS were further acknowledged, with observed reductions in seizure frequency over time (29, 30). Moreover, during the use of invasive VNS in epilepsy patients, the blood concentrations of ASMs remained unaffected by VNS and could even reduce the dosage of ASMs (31, 32). Currently, invasive Vagus Nerve Stimulation has been approved for epilepsy treatment in numerous countries and regions, including the European Union, the USA, Canada, China, and Japan (33). In recent years, although iVNS has shown promising results in the treatment of drug-resistant epilepsy, it is still chosen only after antiseizure medications and surgical options have been ruled out, primarily due to its invasive nature and cost-effectiveness concerns. This is largely due to its invasive nature and cost-effectiveness concerns. Studies on the cost-effectiveness of iVNS have demonstrated a significant reduction in epilepsy-related direct medical expenses following VNS implantation compared to pre-implantation costs (34–36). Evans et al. (37) included the cost of device implantation in their analysis and obtained similar results. However, the reduction in costs is observed only when the treatment is effective and not prematurely discontinued due to ineffectiveness or severe side effects. Additionally, surgical and other medical costs vary across countries, leaving the cost-effectiveness of VNS still uncertain (33).

Further research has found that VNS is effective for various types of epilepsy and can serve as an adjunctive therapy for both focal and generalized seizures. However, in clinical practice, iVNS is more commonly used for patients with focal seizures. Some researchers suggest this could be because focal seizures may develop a higher likelihood of drug resistance, or it might relate to the relatively lower incidence of refractory generalized seizures in adults (33). Recent studies have also discovered positive effects of VNS on drug-resistant generalized seizures, particularly in cases of genetic generalized epilepsy, significantly reducing seizure frequency and epilepsy-related hospitalizations among these patients (38).

Invasive Vagus Nerve Stimulation can also be used as adjunctive therapy for pediatric epilepsy, with abundant research confirming its effectiveness and safety (12, 39). Children’s response to invasive VNS (reduction in seizure rate by ≥50%) is similar to that of adults, and seizure frequency tends to decrease with prolonged treatment (12, 40). Besides reducing seizure rates, iVNS has shown favorable outcomes in seizure duration, severity, post-seizure severity, quality of life, and overall clinical improvement (39–41). iVNS is similarly effective for pediatric or adolescent epilepsy syndromes, including Dravet syndrome (DS), Lennox–Gastaut syndrome (LGS), hypothalamic hamartomas, epileptic encephalopathies, Rett syndrome, and tuberous sclerosis complex (TSC) (39, 42–49). Initially, the FDA approved iVNS for patients aged 12 and older. However, Elliott et al. found no significant differences in treatment efficacy or complications between children under 12 and older children (12 to under 18 years old) (50). The American Academy of Neurology guideline development subcommittee suggested that for children with partial and generalized seizures who are not suitable for brain surgery, iVNS could be considered (29). Subsequent clinical studies reported the use of iVNS in children around 5 years old, revealing more pronounced improvements in quality of life and cognitive outcomes in the younger age group (under 5 years old) (41). Thus, for children with drug-resistant epilepsy, considering VNS implantation early on could mitigate the adverse effects of epilepsy on their development.

Studies have suggested that Vagus Nerve Stimulation can serve as an adjunctive therapy to control seizures during pregnancy for women with epilepsy (51, 52). There are reported cases where expectant mothers undergoing iVNS treatment for epilepsy during pregnancy led to fetal anomalies, including unilateral congenital glaucoma, mild malformations, and heart murmurs (52, 53). However, in these cases of neonatal anomalies, these pregnant women were also on multiple antiseizure medications in addition to VNS therapy. Ding et al. (54) reviewed and analyzed past relevant literature, concluding that VNS during pregnancy is relatively safe and effective for both the fetus and the mother, suggesting there’s no need to deactivate VNS during pregnancy. However, clinical trials involving VNS therapy for pregnant women with epilepsy are scarce, and the sample sizes in existing case reports are small. Additionally, VNS is often used in conjunction with ASMs, making it challenging to distinguish whether adverse reactions are solely attributable to VNS as an isolated factor. Therefore, further relevant experiments are necessary to determine the suitability of VNS for pregnant women.

Determining the optimal stimulation parameters for patients of different ages and those with specific seizure types or syndromes remains uncertain. The side effects and stimulation levels in invasive Vagus Nerve Stimulation might be closely related. Higher stimulation levels tend to result in higher rates and severity of side effects compared to lower stimulation levels (32). The settings recommended by manufacturers for VNS therapy in epilepsy are typically 1.5–2.25 mA with a pulse width of 250 μs and a frequency of 20 Hz. However, manufacturers only provide recommendations for therapeutic dosing and titration rather than definitive guidelines (55). Fahoum et al. attempted to explore target dosing for drug-resistant epilepsy (DRE) and inferred overall target output current and duty cycle for VNS therapy in epilepsy to be 1.61 mA and 17.1% duty cycle, respectively. Their analysis suggested that patients with longer durations of VNS therapy were more likely to respond to treatment, implying that patients with inadequate sustained dosing might still benefit from achieving target dosing. Therefore, improvements in VNS outcomes might be achieved through appropriately implementing evidence-based dosing and titration guidelines (55). Additionally, rapid titration in DRE patients results in faster clinical benefits regardless of age, and children demonstrate greater tolerance to rapid titration (56, 57). However, most of these reports assess efficacy based on response rates. In practice, while considering efficacy, it’s essential to tailor parameters differently for individuals based on their unique constraints (such as age, physique, etc.) and side effects.

Based on existing clinical trials targeting transcutaneous Vagus Nerve Stimulation (tVNS) for treating drug-resistant epilepsy, the majority of scholars widely acknowledge tVNS as an effective, cost-efficient, and relatively safe adjunct therapy for such patients (58–63). These studies assessed the effectiveness of tVNS in terms of seizure frequency, severity, quality of life, and epilepsy rating scales. Additionally, compared to invasive Vagus Nerve Stimulation, research indicates that transcutaneous Vagus Nerve Stimulation exhibits milder adverse reactions (60, 61, 63, 64). tVNS has also shown positive clinical outcomes for post-stroke epilepsy (65). Furthermore, studies have clinically validated the safety and efficacy of transcutaneous auricular Vagus Nerve Stimulation in treating childhood epilepsy (24). These findings suggest that tVNS represents a highly promising adjunct therapy for drug-resistant epilepsy applicable to both adults and children. However, differences exist in the devices used, stimulation parameters, and duration of studies in these clinical trials, hence the optimal stimulation parameters for tVNS remain unclear (66, 67), necessitating further research and exploration. tVNS holds significant potential in epilepsy treatment, given its non-invasive nature and relatively high safety profile, making it more acceptable to patients. However, there is currently no research indicating how to choose between tVNS or iVNS for patients with drug-resistant epilepsy. Apart from their efficacy in treating epilepsy, the choice between iVNS and tVNS in practice also involves cost-effectiveness considerations, which require further supporting evidence.

### 3.2 Depression

In clinical trials of Vagus Nerve Stimulation for epilepsy treatment, an unexpected discovery emerged regarding VNS’s regulatory effect on human emotions, suggesting a sustained improvement in mood (68, 69). These studies revealed the potential of VNS in treating depression. Early studies predominantly involved treatment-resistant depression patients (those unresponsive to various antidepressant therapies, including medications and electroconvulsive therapy). Following VNS treatment, these patients exhibited notable improvements in their 28-item Hamilton Rating Scale for Depression (HRS-D-28), Montgomery-Åsberg Depression Rating Scale (MADRS), and Clinical Global Impression Improvement score (CGI-I1 or 2) (70, 71). Additionally, these patients showed good tolerance to the treatment (72, 73). In subsequent randomized controlled trials, treatment-resistant depression patients receiving adjunctive VNS showed better performance on depression-related assessment scales compared to those undergoing conventional therapy alone, indicating the positive effects of adjunctive VNS (74, 75). Furthermore, apart from clinical efficacy, VNS also enhanced their quality of life, reduced mortality, and suicide rates (5, 75, 76). However, John et al. found in their experiments that the short-term efficacy (10 weeks) of VNS as an adjunctive treatment for treatment-resistant depression was not significant (77). Moreover, other experiments have shown that VNS-induced improvement in symptoms of depression accumulates over time in the short term and persists (5, 75, 78), suggesting that longer treatment durations might be necessary to achieve the desired effects when using VNS for depression. In 2005, VNS was approved for depression treatment. The National Institute for Health and Care Excellence (NICE) released guidelines for the use of invasive Vagus Nerve Stimulation in treating treatment-resistant depression, suggesting its consideration when patients have failed multiple antidepressants and psychological therapies, including electroconvulsive therapy or other forms of neurostimulation, such as VNS. Current studies mostly demonstrate iVNS as an effective anti-depression treatment, although the evidence literature is limited and requires further substantiation of its efficacy and safety.

Additionally, VNS might offer promising prospects for pregnant women and children with depression. Antidepressants have a range of effects on pregnant women, such as miscarriage, premature birth, fetal malformations, and neonatal adaptation syndrome (79). In this context, VNS as a treatment approach provides a new avenue. A case report from 2005 detailed significant relief from pregnancy-related depression in a patient receiving a combination of antidepressants and VNS therapy (80). There have been noticeable improvements in emotional and depression-related assessment scores among pediatric patients as well (81). However, the effectiveness and safety of VNS therapy for treatment-resistant depression in children and pregnant women have not been well elucidated. Therefore, more research is needed to determine specific criteria for selecting candidates for iVNS treatment within depression.

Transcutaneous Vagus Nerve Stimulation is also effective in treating depression. Several clinical trials using tVNS for treatment-resistant depression have demonstrated its effectiveness and safety. Combining tVNS with standard treatment offers greater benefits to patients (23, 82–85). Furthermore, the efficacy of tVNS in treating depression is comparable to that of citalopram (an antidepressant), suggesting that tVNS could not only serve as an adjunctive therapy but also potentially as an alternative treatment (86). However, there is still a lack of high-quality evidence regarding the effectiveness of tVNS for different types and severity levels of depression (87).

### 3.3 Stroke

Ischemic stroke and hemorrhagic stroke are two types of strokes, both with serious consequences that lead to a range of lingering effects impacting patients’ daily lives and social interactions. Vagus Nerve Stimulation serves as an auxiliary method in conventional rehabilitation treatments, displaying promising outcomes in the treatment of ischemic strokes. In recent years, there has been a growing number of animal experiments and clinical trials investigating VNS treatment for ischemic strokes. However, the application of VNS therapy is excluded for hemorrhagic strokes.

#### 3.3.1 Limb motor function

Stroke often leads to loss of limb motor function. Studies indicate that about 60% of patients still experience upper limb movement impairments 6 months after surgery, significantly impacting their quality of life (88). VNS has been widely used in clinical settings for the recovery of upper limb function after ischemic stroke. Numerous clinical trials suggest that VNS in conjunction with rehabilitation (a home exercise program) has a positive impact on upper limb recovery in patients. Measurements such as the Fugl-Meyer Upper Extremity Assessment (FMA-UE), Wolf Motor Function Test (WMFT), Box and Block Test, Nine-Hole Peg Test, Stroke Impact Scale, and Motor Activity Log (MAL) all show positive changes (89–91).

Research has also emerged on tVNS for upper limb function recovery in stroke patients. Compared to a sham-VNS combined with upper limb movement therapy group, the Fugl-Meyer (UFM) scores significantly improved in the non-invasive vagus nerve stimulation combined with upper limb movement therapy group. This demonstrates the feasibility and effectiveness of tVNS in upper limb function recovery (92, 93). Motor-activated auricular vagus nerve stimulation (MAAVNS) is a closed-loop vagus nerve stimulation system that combines electromyography (EMG) and taVNS. Initially, EMG sensors detect movement in a specific muscle, and the EMG signal is then processed to activate the nerve stimulator, pairing muscle movement with tVNS (94). In a recent study, Badran et al. found that MAAVNS was more beneficial for upper limb function recovery (twice as effective as taVNS alone), and the total stimulation pulses received by the MAAVNS group were also fewer than those in the taVNS group (95).

#### 3.3.2 Sensory function

Besides the impairment in limb movement, sensory loss after a stroke is also quite common, and successful movement often relies on the integration of sensory and motor functions (96). In a clinical study focusing on VNS treatment for upper limb movement impairments, a patient experiencing both motor and sensory issues underwent VNS therapy, resulting in significant recovery in limb movement while the sensory problems persisted. Subsequently, this patient received VNS combined with sensory therapy, leading to a marked improvement in sensory function (97). VNS holds promise as a new approach to treating sensory impairments, yet further evidence is needed to support this.

#### 3.3.3 Dysphagia

Stroke patients commonly experience difficulty swallowing, which can lead to several complications such as pneumonia, dehydration, and malnutrition, with pneumonia being a severe complication that can cause fatalities. Yuan et al. reported a case where a patient’s oral swallowing function significantly improved after 6 weeks of using ta-VNS for treating swallowing difficulties (98). A recent double-blind, placebo-controlled, parallel study involving 40 acute stroke patients arrived at similar conclusions (99). tVNS holds promise as a complementary therapy for post-stroke swallowing difficulties, yet further research is needed.

### 3.4 Disorders of consciousness

Disorders of consciousness (DOC) refer to impaired consciousness following severe brain or neurological injury. Based on their neurological behavioral functions, DOC can be classified into four categories: coma, vegetative state/unresponsive wakefulness syndrome (VS/UWS), minimally conscious state (MCS), and emergence from MCS to higher levels of consciousness (eMCS) (100). In recent years, the utilization of vagus nerve stimulation (VNS) techniques has sparked considerable interest among neuroscientists for treating disorders of consciousness. These techniques offer a promising neuroregulatory treatment method for the rehabilitation of DOC patients.

Studies investigating the use of vagus nerve stimulation in treating disorders of consciousness have explored various etiologies, including traumatic brain injury (TBI) (101), stroke (102), hypoxic–ischemic encephalopathy (HIE) (103), and intracerebral hemorrhage (104). Vagus nerve stimulation, whether invasive VNS (105) or non-invasive VNS (101, 103, 106), has shown favorable outcomes in treating DOC. In these studies, assessment of consciousness disorders includes behavioral assessment and brain function evaluation. Behavioral assessment of DOC patients primarily utilizes the revised Coma Recovery Scale-Revised (CRS-R). Nearly all studies using CRS-R as a primary outcome measure reported significant improvements in scores post-intervention (101, 103, 105–107), except for Wang et al.’s study (which did not find significant changes in the scale) (108). Additionally, efficacy might increase over extended periods of time (109). Brain function assessment via scale scoring includes electroencephalograms (EEG) (105), positron emission tomography (PET) (105), and functional magnetic resonance imaging (observed changes in default mode networks and cerebral blood flow) (103, 104), providing evidence of cerebral changes post-stimulation. Yu et al. (104) found that individuals who responded to auditory stimulation (RtAS) had demonstrated better therapeutic outcomes following tVNS compared to those who did not respond, suggesting that preserved auditory function might be a key preexisting factor for tVNS responders among DOC patients.

Research on VNS has primarily focused on patients classified as VS/UWS or MCS. Wang observed significant alterations in cortical connectivity in specific brain regions through electroencephalographic observation in MCS patients, with noticeable changes in interbrain connectivity in MCS patients (108). Noe et al. also found a significant improvement in CRS-R scores in MCS patients with tVNS, while VS/UWS patients did not significantly benefit (101). However, other studies have indicated that VS/UWS patients also benefit from vagus nerve stimulation (104). In summary, the specific target population for the application of VNS in DOC remains inconclusive. In a review of VNS treatment for DOC, the author concluded that existing studies had insufficient sample sizes, low quality (lack of strict adherence to methodology, inadequate sample size selection, reliance on single outcome indicators to assess consciousness levels, and short monitoring follow-up), thus preventing definitive conclusions or recommendations (110).

### 3.5 Alzheimer’s disease

Alzheimer’s disease (AD) typically starts subtly and gradually progresses, manifesting as declining cognitive abilities and memory impairments, eventually affecting normal life (111). There is not extensive research on iVNS (implantable vagus nerve stimulation) for treating Alzheimer’s disease, but the existing reports show promising outcomes. Two reports involving 27 AD patients revealed improvements or stability in Alzheimer’s disease assessment scales such as the Alzheimer’s Disease Assessment Scale-Cognitive Subscale (ADAS-cog), the Mini-Mental State Examination (MMSE), and Clinician’s Interview-Based Impression of Change plus Caregiver Input (CIBIC+) (112, 113). Additionally, patients did not experience a significant decline in mood, behavior, or quality of life 1 year post-treatment (113). Apart from enhancing cognitive capabilities, iVNS demonstrates a positive impact on memory loss, effectively ameliorating Alzheimer’s disease symptoms. Studies indicate that iVNS enhances word recognition memory, working memory, and memory retention in language learning (114, 115). In summary, iVNS may delay AD progression and improve its symptoms. However, reports on iVNS treatment for AD are limited and have garnered little attention. This might be attributed to the fact that AD patients are mostly elderly, and the high risk of invasive vagus nerve stimulation in the elderly population cannot be overlooked.

The impact of tVNS on Alzheimer’s disease is currently under exploration in human trials (NCT04908358, NCT04877782). One study involving 140 elderly individuals will divide participants into tVNS and sham stimulation groups, receiving percutaneous vagus nerve stimulation-breathing controlled auricular electrical stimulation for 10 min (twice, with a 4-week interval). The efficacy of VNS will be determined through the Face-Name Associative Memory Exam (FNAME) and Alzheimer’s disease-related biomarkers in blood. Another study aims to explore the impact of tVNS on memory and attention by monitoring its effects on Alzheimer’s disease.

Research on the effects of tVNS on memory has also emerged. Early findings suggested that tVNS could alter associative memory performance in older individuals (116). In an experiment involving 60 healthy volunteers, tVNS did not impact word processing or overall emotional memory recognition. However, compared to sham stimulation, tVNS increased the hit rate percentage for high confidence memory words, suggesting an impact on recollective memory performance (117). Sun et al. proposed that tVNS has beneficial effects on offline working memory in healthy individuals (118). Mertens attempted to replicate the positive effects of iVNS on word recognition memory using tVNS but found no improvement in word recognition memory in healthy individuals (119). Researchers speculated that this outcome might be related to the parametric design of the stimulation. Mariana compared Mertens’ parameter settings with those used in other papers that measured successful memory outcomes, indicating that the standard “off” interval length might be critical for modulating successful memory (120). Recent studies also suggest that tcVNS can improve attention, performance, and working memory (121).

There’s substantial research on the effects of tVNS on cognition, demonstrating that electrical stimulation can enhance cognitive abilities and increase cognitive control in healthy volunteers (122–124). Studies found that tVNS modulates conflict-induced cognitive control and action execution in behavioral and electroencephalogram data (125). There are varying perspectives on the impact of tVNS on cognitive flexibility. Tona et al. suggested that tVNS does not influence working flexibility (126). However, Borges remains optimistic about the potential of tVNS to influence cognitive flexibility. In his study, he compared the effects of tVNS on core executive functions, including inhibitory control and cognitive flexibility, and suggested that tVNS might have a stronger impact on cognitive flexibility than on inhibitory control (127). In recent research, tVNS has shown promising implications for neurocognitive functional recovery in elderly patients, effectively reducing the occurrence of delayed neurocognitive recovery (dnCR) after total joint arthroplasty (TJA) in the elderly (128). Effective results were observed in cognitive impairment patients by stimulating two ear acupoints related to the heart (auricle CO15) and kidney (CO10) (129). This provides evidence for tcVNS in slowing cognitive decline in the elderly and preventing dementia.

As Alzheimer’s disease is common among the elderly, the risk of carotid atherosclerotic plaques is relatively higher due to its specific nature (111). Vargas-Caballero et al. (120) mentioned in their article that the surgical implantation of iVNS devices into the cervical vagus nerve would involve manipulating the carotid arteries, increasing the risk of plaque rupture and leading to severe complications like cerebrovascular disease. While invasive vagus nerve stimulation and its potential for greater closed-loop interventions might be preferable clinically, non-invasive vagus nerve stimulation may be a better choice for treating Alzheimer’s disease (120). tVNS demonstrates certain effects on memory capabilities and cognition, has low costs, and high safety, making it a very promising treatment for Alzheimer’s disease, which mainly affects elderly patients. However, the effectiveness of tVNS as a treatment method requires further clinical trials.

### 3.6 Anxiety

VNS has been shown to alleviate anxiety symptoms, a finding initially observed in epilepsy patients. In addition to improving epilepsy and depression, iVNS has been found to significantly reduce anxiety levels (130). George et al. (131) reported that iVNS demonstrated notable efficacy in treatment-resistant obsessive-compulsive disorder (OCD), panic disorder, and post-traumatic stress disorder (PTSD) based on Hamilton Anxiety Rating Scale (HAM-A) scores. Furthermore, OCD patients showed significant improvements in Yale-Brown Obsessive-Compulsive Scale (Y-BOCS) scores. Additionally, iVNS has been found beneficial in alleviating PTSD symptoms, effectively enhancing the extinction of conditioned fear (132, 133). The anxiolytic effects of iVNS appear to be frequency-dependent (134). In studies by Burger et al. (135), tVNS also accelerated fear extinction but did not lead to better retention of extinction memory after 24 h. However, Genheimer et al. (136) presented contrasting findings, as her study did not observe significant anxiety reduction with tVNS. Therefore, further research is necessary to clarify these effects.

### 3.7 Autism spectrum disorder

Initially, VNS was found to alter the behavior of children with epilepsy and ASD, including changes in alertness, mood, and communication styles. It also significantly improved various aspects of quality of life (137–139). Moreover, these behavioral and developmental improvements were found to be independent of epilepsy control, further demonstrating the potential of VNS in treating ASD (140). However, research related to ASD remains highly limited, and whether VNS provides benefits for children with ASD is still an open question.

### 3.8 Parkinson’s disease

In a study using nVNS to treat gait disturbances associated with Parkinson’s disease (PD), tcVNS improved gait parameters and alleviated freezing of gait (FOG) symptoms in PD patients. Significant reductions were observed in UPDRS III scores, with notable improvements in gait parameters such as walking speed, stride length, and step velocity (141). The number of steps required for turning was particularly reduced. Additionally, Farrand et al. (142) found that higher VNS frequencies contributed to behavioral improvements and attenuation of pathological markers in PD models. These findings suggest that VNS may be a potential therapeutic option for PD, although further studies are required to validate and optimize its application in PD treatment.

### 3.9 Migraine and cluster headache

Since some epilepsy patients also experience migraine symptoms, iVNS was initially found to improve migraine symptoms while alleviating epilepsy (143). Hord et al. (144) followed up with epilepsy patients undergoing iVNS treatment and similarly received positive feedback regarding iVNS’s efficacy in relieving migraine. Subsequently, iVNS was further demonstrated to be effective in alleviating chronic refractory migraine and cluster headache, although these studies involved small sample sizes (145, 146). Compared to iVNS, nVNS has attracted more attention. Both tcVNS and taVNS have been found to significantly relieve acute migraine, reduce headache days and attack frequency, lower pain intensity, and improve pain-free rates (147, 148). Studies on chronic cluster headache have shown that tcVNS can terminate pain within 11 ± 1 min (149). Additionally, nVNS has shown promising results in relieving acute cluster headaches, with sustained treatment potentially providing further benefits. However, in chronic cluster headache cohorts, tcVNS has not demonstrated significant advantages (150). Although increasing evidence suggests that VNS could bring significant benefits to patients with migraine or cluster headache, further high-quality studies are urgently needed.

## 4 Mechanisms of VNS in CNS disorders

Vagus nerve stimulation therapy demonstrates intricate mechanisms in its application for treating central nervous system disorders. Presently, the mechanisms of VNS in central nervous system disorders remain inconclusive, and research into its mechanisms is an ongoing exploration. This holds significant importance in expanding VNS applications within the central nervous system clinically. The mechanisms of VNS are associated with the anatomy of the vagus nerve. Vagus nerve fibers have four projection areas in the brainstem, including the nucleus ambiguus (NA) and dorsal motor nucleus (DMV) for the efferent branches of the vagus nerve, along with the nucleus tractus solitarii (NTS) and spinal trigeminal nucleus for incoming fibers (151). NTS acts as the primary relay station for vagus nerve input in the brain, receiving the most incoming vagus nerve signals. Moreover, this nucleus projects to various structures, such as periventricular gray matter, dorsal raphe nucleus, paraventricular nucleus of the thalamus, amygdala, and septum. When the vagus nerve receives external electrical stimulation, the incoming fibers get excited, and the incoming signal spreads from peripheral nerves to the NTS and locus coeruleus (LC), eventually propagating to subcortical structures (mainly the hippocampal region) and cortical structures (including the insular cortex, prefrontal cortex, and motor cortex) (152). Previous research analyses suggest that VNS primarily functions by inhibiting central inflammation, promoting neuroprotection, and enhancing cortical plasticity integrity.

### 4.1 Suppression of central inflammation

**Figure 2:**
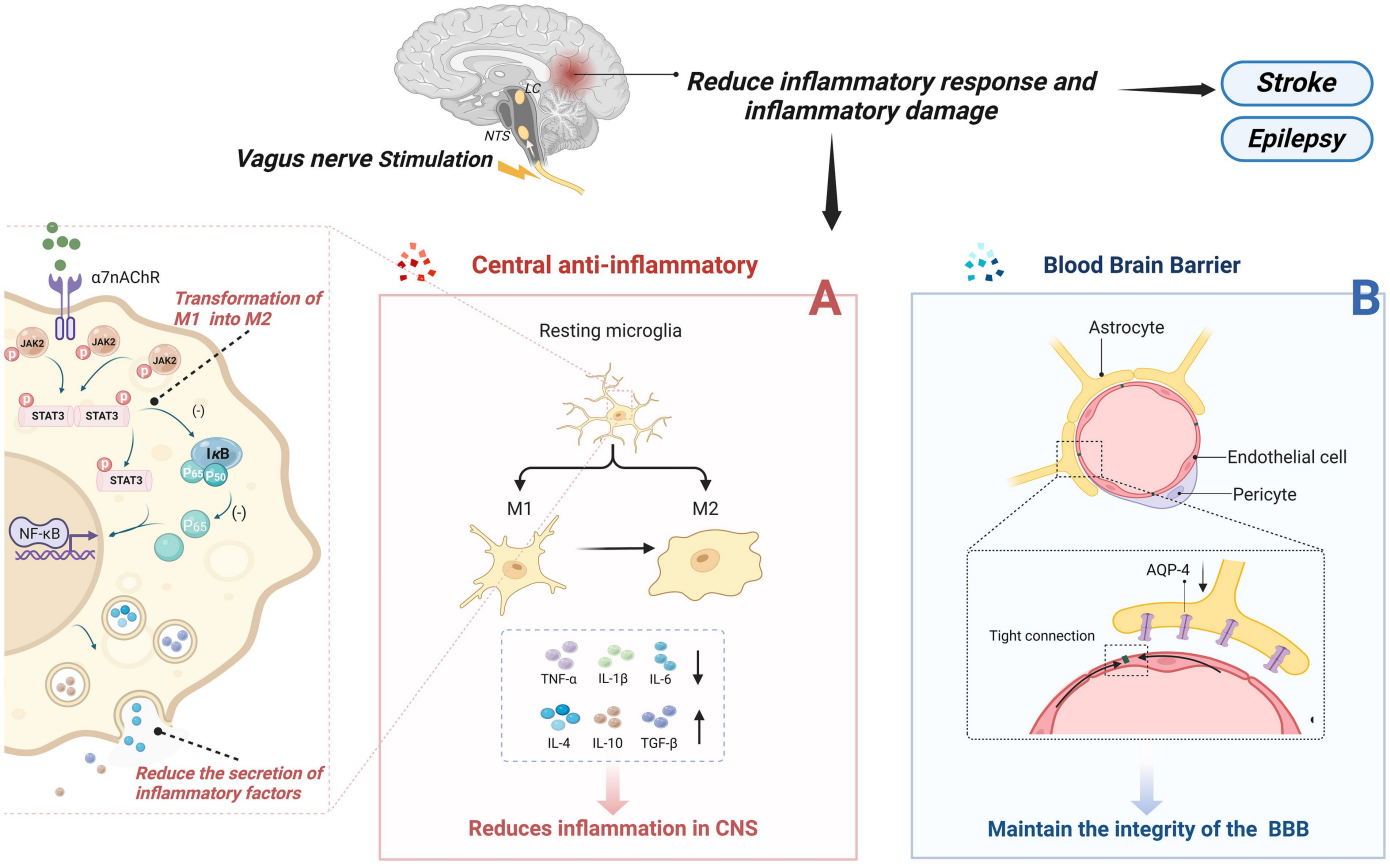
Central anti-inflammatory effect of VNS. **(A)** VNS changed microglia phenotype from M1 phenotype to M2 type; On the other hand, by activating the a7nAchR receptor, which is widely present in the CNS, the inflammatory cytokines secreted by pro-inflammatory cells are reduced. **(B)** Maintain the integrity of the blood-brain barrier and prevent the destruction of the central nervous system by inflammatory factors or other substances.

Inflammation is a protective response of the body to external stimuli; however, excessive inflammation can lead to structural damage in brain function. Neuroinflammation is triggered by activated and proliferating microglia, astrocytes, and other myeloid cells, which produce pro-inflammatory cytokines, chemokines, and other inflammatory mediators, ultimately leading to neuronal damage (153). When a stroke occurs, a severe inflammatory response in the central nervous system damages neurons, and VNS’s central anti-inflammatory mechanism can alleviate this process. Several central nervous system diseases are closely associated with the occurrence of excessive inflammation, such as epilepsy and depression (154–156). The central anti-inflammatory effect of VNS has drawn significant attention and research from many scholars, with abundant and compelling evidence demonstrating its inhibitory role in inflammation. Its mechanisms primarily encompass three aspects (Figure 2): firstly, it can alter the phenotype of microglia cells; secondly, it reduces the secretion of inflammatory cytokines by activating the widespread α7nAchR receptors present in the CNS; and finally, it maintains the integrity of the blood–brain barrier, preventing inflammatory factors from damaging the central nervous system. The α7nAchR receptor is a crucial anti-inflammatory participant in the human body and is extensively expressed in the brain, including neurons, neuroglial cells, and endothelial cells. Activation of these receptors enhances the neurons’ resistance to ischemic or other types of injuries (157). Moreover, research indicates that VNS can induce an endogenous cholinergic anti-inflammatory pathway by increasing acetylcholine release through vagus nerve activation, subsequently activating α7nAchR receptors upon binding with acetylcholine (158).

#### 4.1.1 Alteration of microglial cell phenotype

As part of the homeostasis of the central nervous system, resting microglia maintain immune surveillance under physiological conditions. They have the ability to engulf pathogens and cellular debris that invade the brain and, under certain stimuli, transform into an activated phenotype to perform their function (159). Activated microglia undergo morphological changes and primarily exist in two phenotypes: the M1 phenotype, which secretes pro-inflammatory factors and induces self-perpetuating damage to neurons; and the M2 phenotype, which protects neurons and promotes recovery (160). Chen et al. found that tVNS induces a shift in the phenotype of microglial cells from the M1 polarized Iba-1/CD86 microglia to the M2 polarized Iba-1/CD206 microglia (161), enhancing the anti-inflammatory capacity of microglial cells. Additionally, the α7nAchR is identified as a critical target for altering the phenotype of microglial cells. In an Alzheimer’s disease model, tcVNS significantly shifted the phenotype of microglial cells from neurodestructive to neuroprotective, increasing the release of BDNF, basic fibroblast growth factor (bFGF), anti-inflammatory cytokines (IL-4, IL-10, TGF-β), and decreasing the release of pro-inflammatory cytokines (IL-1β, IL-6, TNF-α), ultimately delaying cognitive decline (162). Zhang et al.’s research (163) suggests that the JAK2/STAT3 pathway might be a downstream pathway of α7nAChR. Activation of α7nAChR by ACh through this pathway promotes the transformation of M1 microglial cells into the M2 phenotype.

#### 4.1.2 Reduction of the secretion of inflammatory cytokines

Reducing Inflammatory Factors with VNS is also related to the activation of α7nAchR on microglial cells. The activation of α7nAchR on microglial cells may provide neuronal protection *in vitro* under conditions of hypoxia and glucose deprivation, reducing the inflammatory response to ischemic damage (164). Studies have shown that after 24 h of vascular occlusion in I/R mice, VNS activated α7nAchR on microglial cells, reducing levels of inflammatory cytokines (TNF-α, IL-1β, and IL-6), while accompanied by an increase in anti-inflammatory cytokines (IL-4, IL-10, TGF-β), thus safeguarding neuronal functional recovery following acute brain injury (162, 165, 166). Lu et al. (167) suggested that VNS’s post-stroke anti-inflammatory effect might involve the activation of α7nAChR on microglial cells, amplifying the levels of phosphorylated signal transducer and activator of transcription 3 (p-STAT3) and Janus kinase 2 (JAK2). Increased phosphorylation of both inhibits the NF-κB pathway, reducing the production of pro-inflammatory cytokines (168). In a mouse model of brain ischemia, Jiang et al. found that VNS might upregulate the expression of peroxisome proliferator-activated receptor gamma [PPARγ, a ligand-activated transcription factor that plays a positive anti-inflammatory and neuroprotective role during CNS inflammation (169)] by activating α7nAChR, thereby suppressing inflammatory factors in the CNS and exerting an anti-inflammatory effect (170). Further evidence supports the crucial role of α7nAChR in VNS’s reduction of inflammatory factors. Similarly, tVNS can upregulate the expression of α7nAchR on hippocampal microglial cells. α7nAchR prevents the expression of phosphorylated-p65 and nuclear translocation of the NF-κB pathway, thereby regulating the expression of pro-inflammatory cytokines, alleviating depressive symptoms (171).

#### 4.1.3 Maintenance of blood–brain barrier integrity

Maintaining the integrity of the blood–brain barrier (BBB) is crucial for the stability of the central nervous system (CNS) as it effectively prevents certain substances, mostly harmful, from entering the brain from the bloodstream, thus playing a pivotal role in mitigating CNS inflammatory reactions post-stroke (172). Additionally, increased BBB permeability is often associated with inflammation-related neurodegenerative changes (173). Current research suggests that Vagus Nerve Stimulation can improve the integrity of the BBB, reducing its permeability, thereby preventing inflammatory factors from entering the CNS, effectively alleviating symptoms in mice with cortical microinfarcts (CMI) (174). However, the specific mechanisms by which VNS regulates BBB permeability remain unclear. Some researchers suggest that Vagus Nerve Stimulation lowers brain vascular permeability, possibly by preventing the upregulation of Aquaporin-4 (AQP-4) around blood vessels (175). Others propose the presence of nerve terminals around the BBB (potentially including cholinergic and noradrenergic terminals), and activation of α7nAchR may reduce BBB permeability. This suggests that VNS might transmit signals to the BBB through these peripheral cholinergic and noradrenergic terminals (176). Another potential mechanism involves immune regulation in the brain microenvironment. VNS could reduce the expression of Matrix Metalloproteinases-2/9 (MMP-2/9) in astrocytes while safeguarding vascular endothelial cadherin from microvascular damage (177). Jin et al. (178) proposed a new hypothesis stating that VNS enhances the concentration of neurotrophic factors within the brain (such as BDNF), potentially serving as a protective mechanism for the BBB.

### 4.2 Promotion of neuroprotection

**Figure 3:**
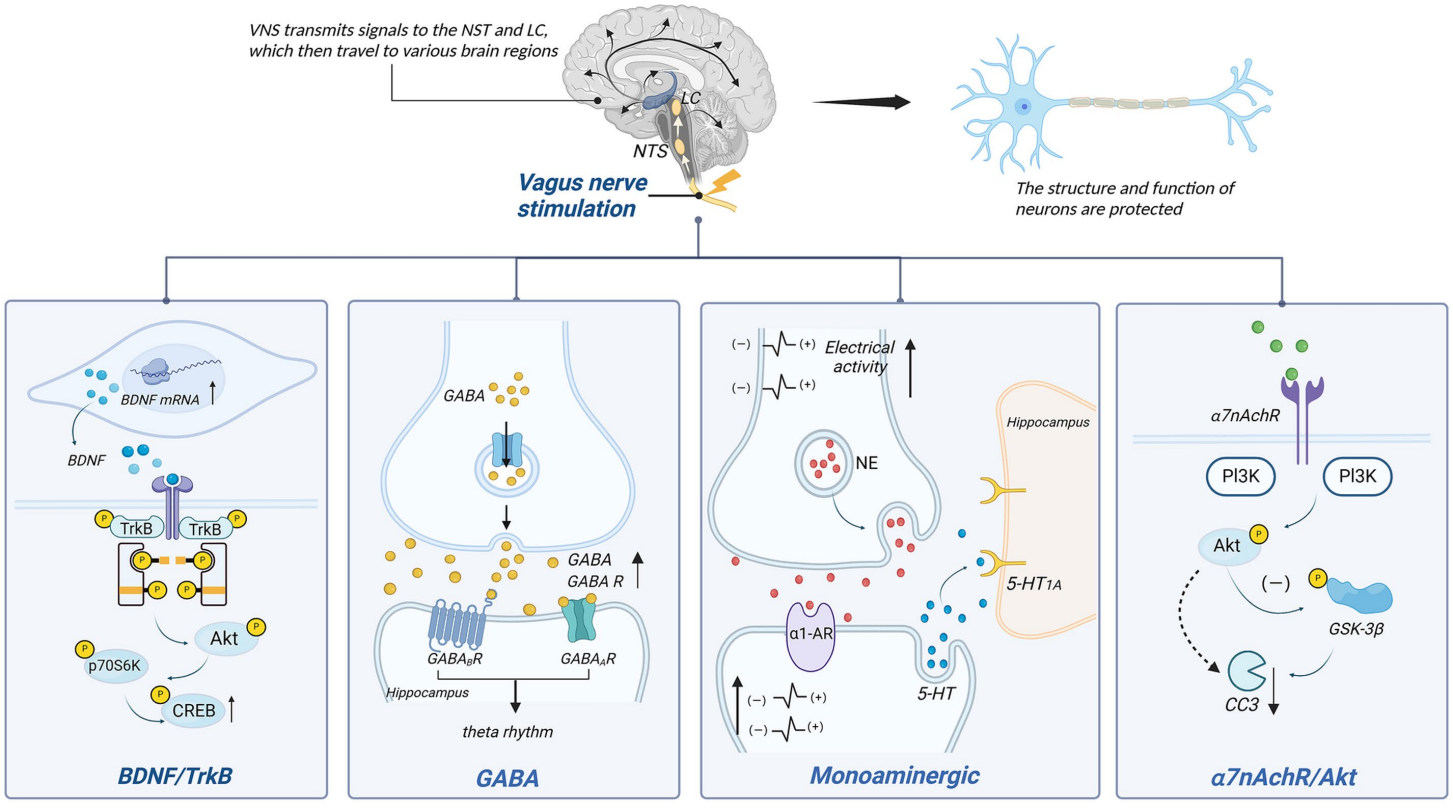
Neuronal protection of VNS. p70S6K, p70 ribosomal protein S6 kinase kinases; CREB, cAMP-responsive element-binding protein; PI3K, phosphatidylinositol 3-kinase; NE, norepinephrine; α1-AR, α1-norepinephrine receptors; GSK-3β, glycogen synthase kinase-3β; CC3, cleaved caspase 3.

Previous research indicates that the vagus nerve plays a significant role in neuroprotection (Figure 3). Various neuroprotective pathways of Vagus Nerve Stimulation (VNS) have been discovered, including the release of neurotrophic factors, modulation of neurotransmitters, and improvement in cerebral vascular regeneration.

#### 4.2.1 BDNF/TrkB

Vagus Nerve Stimulation therapy for depression is closely linked to the BDNF/TrkB signaling pathway. BDNF, a brain-derived neurotrophic factor, protects neurons by enhancing synaptic plasticity, inhibiting cell apoptosis, and promoting nerve regeneration. Tyrosine receptor kinase B (TrkB) serves as the primary receptor for BDNF. The BDNF/TrkB signaling pathway has been shown to be closely associated with the occurrence and development of various brain disorders (179). In individuals with mood disorders, notably, BDNF levels are significantly reduced, while antidepressants can increase BDNF expression and signaling (180). The mechanism behind VNS’s effect on depression may be linked to this signaling pathway.

Studies have indicated a close relationship between the vagus nerve and BDNF. When the vagus nerve is severed, hippocampal BDNF mRNA expression decreases, leading to diminished neuronal changes (181). Research by Follesa et al. (182) demonstrated increased BDNF expression in the hippocampus and cerebral cortex of rats after just 3 h of VNS. This effect is also observed in chronic VNS, where the hippocampal cell proliferation increases, and there are sustained alterations in neuronal dendritic complexity (183). Recent findings suggest that when α7nAchR is blocked, the activity in the BDNF pathway is inhibited, supporting the notion that α7nAchR might mediate VNS’s effects on BDNF (184).

TrkB also plays a role in this context. Following vagus nerve stimulation, phosphorylation occurs at tyrosine 705, 816, and 515 on the hippocampal TrkB receptor. Blocking the TrkB receptor significantly diminishes or even eliminates the therapeutic effects of VNS (185). Phosphorylation at tyrosine 705 facilitates the regulation of other tyrosines and their activation (186). Phosphorylation at tyrosine 515 leads to the activation of Ras/MAPK and PI3K signaling pathways, while phosphorylation at tyrosine 816 induces survival signal transduction mediated by phospholipase C-γ1 (PLC-γ1), inducing neuroprotection (187). Carreno et al. (188) further identified downstream factors of Y515, including Akt and p70 ribosomal protein S6 kinase (p70S6k), ultimately resulting in the phosphorylation of cAMP-response element-binding protein (CREB). Additionally, experimental evidence has confirmed the ligand-dependent activation of TrkB (189). This indicates that both BDNF and TrkB are indispensable components in the VNS mechanism for treating depression, and the BDNF/TrkB signaling pathway plays a crucial role in VNS’s antidepressant effects.

#### 4.2.2 Monoaminergic neurotransmitter

Monoamine neurotransmitters encompass norepinephrine (NE), dopamine (DA), and serotonin (5-HT). The mechanism by which VNS improves treatment-resistant depression might be associated with increased baseline discharge activity of norepinephrine (NE) neurons in the LC, enhancing noradrenergic output (190). It’s also related to the increase in 5-HT in the dorsal raphe nucleus (DRN) after 14 days (191). This effect is similar to the desensitization of the 5-HT1A autoreceptor observed after antidepressant use, which restores the normal firing rate of 5-HT neurons in the DRN (192). Moreover, VNS’s anti-anxiety and antidepressant effects rely mainly on 5-HT neurons (193). However, changes in 5-HT1A autoreceptor sensitivity have not been observed after VNS, suggesting that its impact on 5-HT neuron discharge is indirect (154).

Studies suggest that NE might act as an intermediary, enhancing 5-HT neuron discharge by increasing excitability via α1-adrenergic receptor stimulation in the DRN (154). Liu et al. observed diminished efficacy of VNS when noradrenergic neurons were inactivated, further supporting the role of NE as an intermediary (194). Although VNS notably increases extracellular 5-HT levels in the DRN, similar changes were not observed in two postsynaptic structures, the hippocampus and prefrontal cortex (195). Nonetheless, VNS has been shown to enhance the straight activation of 5-HT1A postsynaptic receptors in these regions, likely contributing to augmented serotonergic neurotransmission in the forebrain (154). Research indicates that VNS exhibits antidepressant effects by increasing NE concentrations in the periphery, thalamus, and cortical regions (196). Manta et al. (195) found that long-term VNS not only affects extracellular 5-HT levels but also increases extracellular DA levels detected in the prefrontal cortex (PFC) and nucleus accumbens (NAc), potentially aiding in alleviating depressive symptoms.

VNS’s inhibition of epileptic seizures is also linked to hippocampal and cortical NE concentrations (197). By activating NE neuron excitability, VNS significantly increases NE release in LC projection areas (including the hippocampus and prefrontal cortex) (182), impacting the diminished VNS-induced seizure inhibition. Recent studies corroborate this view, demonstrating that VNS, by increasing activity in noradrenergic neurons in the LC, elevates arousal and causes widespread, uneven cortical activation in epilepsy patients, influencing their sleep–wake states (198). This NE-dependent modulation is likely mediated by α-adrenergic receptors since their activation can suppress epileptiform activity. In a model of pentylenetetrazole-induced seizures in rats, blockade of α2-adrenergic receptors in the hippocampus reversed VNS’s attenuating effect on seizures (197). Recent mouse experiments also underscore the importance of α-adrenergic receptors in inhibiting seizures (199).

#### 4.2.3 GABA

GABA, an inhibitory neurotransmitter, plays a crucial role in the overall control and fine-tuning of excitatory transmission and is associated with various brain disorders (200). It is primarily distributed in the nucleus tractus solitarii (NTS), dorsal motor nucleus of the vagus nerve (DMV), medial septum (MS), and hippocampus (200, 201).

The NTS is a primary medullary site where vagus nerve afferents terminate. Vagus nerve stimulation can enhance GABA release by activating the NTS (202). The GABA system plays a significant role in epilepsy treatment, as observed in patients receiving VNS, where there is a notable increase in total GABA and free GABA levels in the cerebrospinal fluid (203). Within the NTS, γ-aminobutyric acid operates through GABA receptors for inhibitory control. VNS increases inhibition of NTS output via GABA transmission, reducing susceptibility to chemically induced reflex epileptic seizures (204). Marrosu et al. (205) suggested that VNS plays a role in modulating cortical excitability related to epilepsy, associated with the normalization of cortical GABAA receptor density.

Vagus nerve stimulation might impact memory by enhancing neuroplasticity in brain structures associated with memory storage (206). Studies link this memory processing to increased excitability in hippocampal neuron networks and the presence of local theta rhythms (207, 208). Broncel’s research indicated that GABAA and GABAB receptors in the MS (medial septum, a candidate for vagus nerve input from the NTS to hippocampal structures) are involved in regulating hippocampal theta rhythms induced by VNS, closely associated with anxiety behaviors and learning-memory capabilities (208).

Thus, vagus nerve stimulation influences the central nervous system through the GABA system. Additionally, consistently low GABA levels have been found in brain regions responsible for emotional cognitive processes in depression patients, such as the prefrontal cortex, posterior cingulate cortex, anterior cingulate cortex, and amygdala (209). Therefore, it’s speculated that part of VNS’s therapeutic effects on depression might involve the GABA system.

#### 4.2.4 α7nAchR/Akt

Akt, also known as protein kinase B, has diverse roles in the nervous system, playing a crucial part in cell development, function, and survival processes. Phosphorylated Akt (p-Akt) can activate downstream cysteine aspartate-specific proteases, reducing cleaved caspase-3 (CC3), thereby exerting a cell-protective effect (210). Studies by Krafft et al. (211) demonstrated that in a mouse model of cerebral hemorrhage, activation of α7nAchR reduces neuronal cell death by increasing p-Akt and lowering CC3 expression. Moreover, in a mouse model of cerebral ischemia, VNS-induced α7nAchR activation increased p-Akt while reducing cleaved caspase expression in the brain, ultimately reducing neuronal cell death (165). Furthermore, Akt phosphorylation depends on the phosphoinositide 3-kinase (PI3K) signaling pathway (212). Vagus nerve stimulation has been shown to decrease cardiomyocyte apoptosis through the PI3K/Akt signaling pathway (213), indirectly indicating that PI3K may serve as an intermediary in the α7nAchR/Akt pathway. However, further related research is required to confirm this relationship.

### 4.3 Increase of cortical plasticity

Vagus nerve stimulation has been confirmed to produce widespread excitatory effects in various cortical regions, prompting exploration into induced cortical neuroplasticity. In the sensory and motor systems, temporarily pairing VNS with sensory stimulation or movement reorganizes neural circuits associated with sensation and movement networks (214). The cholinergic system in the basal forebrain (214, 215) and activation of the noradrenergic system in the locus coeruleus (216) may play important roles in this neural circuitry reshaping. This involves increased activity in noradrenergic neurons in the LC and cholinergic neurons in the basal forebrain (BF), which are a source of cortical-projecting cholinergic neurons (198, 217). VNS induces acetylcholine release in the basal forebrain and modulates neural activity in the BF, playing a critical role in the cortex (215). Impaired cholinergic projection in the basal forebrain cortex leads to a loss of motor network plasticity (218), while excitability and synchrony in the auditory cortex are disrupted upon blocking muscarinic receptors in the auditory cortex (219).

The sensory system comprises two pathways: the feedback (FB) and feedforward (FF) pathways. The superficial layers of the primary sensory cortex act as the hub for the feedforward (FF) pathway, while the deeper layers serve as the hub for the feedback (FB) pathway. Acetylcholine and norepinephrine, respectively, modulate the FF and FB pathways (220, 221). In the study by Kumagai et al. (199), VNS primarily activated the FF pathway within the sensory system rather than the FB pathway. This indicates that VNS-induced neural regulation can alter the FF-FB balance in the auditory cortex, known as neural gain, and the regulation of FF-FB balance induced by VNS may contribute to various clinical outcomes.

## 5 Pupil dilation may be a sensitive readout of VNS effects on brain states

In the cortex, the activity of basal forebrain cholinergic and local noradrenergic axons can be sensitively tracked by pupillary dilation (222). Furthermore, under constant brightness, pupillary dilation can read out the brain state characteristics of the mouse cortex and hippocampus sensitively and non-invasively (223). As a result, some scholars propose pupillary dilation as a sensitive biosensor for the titration effects of VNS-induced brain neuromodulation states. The amplitude of VNS-induced pupillary dilation corresponds to its stimulation parameters, and this VNS-induced pupillary dilation is mediated by acetylcholine released from the basal forebrain to the neocortex network, interacting nonlinearly with the current momentary brain state (214). However, there is still limited research in this area, and whether pupillary dilation is the most suitable representation of VNS stimulation is yet to be determined. Nevertheless, it’s a promising avenue to explore and provides direction for research on vagus nerve stimulation.

## 6 Conclusion

In conclusion, VNS is a promising therapeutic approach for central nervous system disorders. In addition to its officially approved applications for the treatment of refractory epilepsy and depression, accumulating evidence suggests that VNS may also provide benefits to patients with Alzheimer’s disease, stroke, disorders of consciousness, anxiety, Parkinson’s disease, autism spectrum disorder, migraines, and cluster headaches. Both iVNS and tcVNS can stimulate the vagus nerve (VN) at the carotid sheath and have demonstrated promising results in the treatment of various CNS diseases. However, the effects of taVNS, such as in anxiety disorders, remain controversial and certainly require further research for validation. Furthermore, research should be refined and personalized to address the specific pathological mechanisms of different CNS diseases. Each patient’s bio-psycho-social background should be considered, as this helps provide a more comprehensive and effective treatment strategy. Recent studies indicate that VNS predominantly affects the CNS by inhibiting central inflammation, promoting neuroprotection, and enhancing cortical plasticity. Altering the phenotype of CNS microglial cells, reducing pro-inflammatory factors, and maintaining blood–brain barrier permeability can suppress the intensity of CNS inflammation, thereby reducing damage to brain tissues. Protection of neurons is mainly mediated through modulation of neurotrophic factors or neurotransmitters. However, there are still several challenges in this field. Firstly, VNS often serves as an adjunct or complementary therapy for CNS diseases, and the quality of related literature varies significantly, including differences in sample size and observed indicators. Moreover, optimal stimulation protocols for specific CNS diseases or diverse populations are scarce, limiting its broader application and clinical efficacy. Expanding the indications for VNS and determining the best stimulation protocols require more high-quality research. Secondly, predicting the effectiveness of VNS for various CNS diseases is in its infancy, which poses barriers to targeted treatment. Finally, the precise mechanism of VNS’s therapeutic effect remains unclear. Existing research mostly focuses on the factors influenced by VNS rather than investigating its primary action, severely limiting the expansion of VNS into other CNS diseases. Understanding how VNS interacts with neural circuits could identify new stimulation targets. Therefore, future VNS research will prioritize addressing these issues.

## Glossary

α7nAChR: α7 Nicotinic Acetylcholine Receptor
ABVN: Auricular Branch of the Vagus Nerve
AD: Alzheimer’s Disease
ADAS-cog: Alzheimer’s Disease Assessment Scale-Cognitive Subscale
Akt: Protein Kinase B
AQP-4: Aquaporin-4
ASD: Autism Spectrum Disorder
ASMs: Antiseizure Medications
BBB: Blood–brain barrier
BDNF: Brain-Derived Neurotrophic Factor
bFGF: Basic Fibroblast Growth Factor
CGI: Clinical Global Impression
CIBIC+: Clinician’s Interview-Based Impression of Change
CNS: Central Nervous System
CRS-R: Coma Recovery Scale-Revised
DA: Dopamine
DOC: Disorders of Consciousness
DRN: Dorsal Raphe Nucleus
DRE: Drug-resistant Epilepsy
EEG: Electroencephalograms
FDA: Food and Drug Administration
FNAME: Face-Name Associative Memory Exam
FOG: Freezing of Gait
HAM-A: Hamilton Anxiety Rating Scale
HRS-D-28: 28-item Hamilton Rating Scale for Depression
JAK2: Janus Kinase 2
LC: Locus Coeruleus
MADRS: Montgomery-Åsberg Depression Rating Scale
MCS: Minimally Conscious State
eMCS: Emergence from MCS to higher levels of consciousness
MMP: Matrix Metalloproteinases
MMSE: Mini-Mental State Examination
MS: Medial Septum
NAc: Nucleus Accumbens
NE: Norepinephrine
NICE: National Institute for Health and Care Excellence
NTS: Nucleus Tractus Solitarii
OCD: Obsessive-Compulsive Disorder
PD: Parkinson’s Disease
PET: Positron Emission Tomography
PFC: Prefrontal Cortex
PI3K: Phosphoinositide 3-Kinase
PTSD: Post-Traumatic Stress Disorder
TrkB: Tropomyosin Receptor Kinase B
UPDRS III: Unified Parkinson’s Disease Rating Scale, Part III
VNS: Vagus Nerve Stimulation
iVNS: Implantable Vagus Nerve Stimulation
nVNS: Non-invasive Vagus Nerve Stimulation
tVNS: Transcutaneous Vagus Nerve Stimulation
taVNS: Transcutaneous Auricular Vagus Nerve Stimulation
tcVNS: Transcutaneous Cervical Vagus Nerve Stimulation
VS/UWS: Vegetative State/Unresponsive Wakefulness Syndrome
WMFT: Wolf Motor Function Test
Y-BOCS: Yale-Brown Obsessive-Compulsive Scale
